# Supplementary material for: Adapting Agriculture to Climate Change: A Synopsis of Coordinated National Crop Wild Relative Seed Collecting Programs across Five Continents
Source: Plants (Basel). 2022 Jul 13;11(14):1840. doi: 10.3390/plants11141840 (PMC9319254; doi:10.3390/plants11141840)
Supplement: Supplementary file 1 [file plants-11-01840-s001.zip › Eastwood et al. Supplementary Method S1.pdf]

Supplementary Method for

Title: Adapting Agriculture to Climate Change: A Synopsis of Coordinated National Crop Wild Relative Seed Collecting Programs Across Five Continents

Authors: Ruth J. Eastwood, Beri B. Tambam *et al*.

CWR Project Field Data Form

Collection Number	Collection date	Accession ID

Main collector surname, first name	Institution *(CAPITALS)*
Other collectors names	Institutions

Family	Genus	Species	Subsp.<br>Var.

Vernacular name(s) (+ language)

Photograph IDs or reference numbers

Herbarium voucher Yes/No Number:<br>Soil sample Yes/No Number:<br>Sampling area (m x m)	Number of mature plants found<br>1000+<br>100-999<br>50-99<br>25-99<br>10-24<br>5-9<br>2-4<br>1<br>If counted actual number_______	Number of plants sampled<br>1000+<br>100-999<br>50-99<br>25-99<br>10-24<br>5-9<br>2-4<br>1<br>If counted actual number_______	Sampling method<br>Random<br>Regular<br>Transect (linear)<br>Core of population<br>Edge of population<br>Other	Seeds / fruits collected from ground?<br>Yes No Partially<br>Population distribution<br>Single plant<br>Patchy<br>Continuous	Phenology status<br>More flowers than fruits<br>More fruits than flowers<br>Only fruits<br>Fruits already dispersed

Country	Primary subdivision
Secondary subdivision	Tertiary subdivision
Local area	Locality *(including distance and direction from nearest village)*

Latitude	Longitude	Units<br>Degrees<br>Meters

Altitude (m)	Altitude accuracy (m)	Water depth (m)

Status<br>Weedy<br>Cultivated<br>Wild<br>- - - - -<br>Native<br>Introduced<br>Naturalised	Lat/Long method<br>GPS<br>(Datum_________)<br>DGPS<br>Map<br>Google Earth	Altitude method<br>Altimeter<br>DEM<br>GPS<br>Estimate<br>Map	Prevalent aspect<br>N<br>N-E<br>E<br>S-E<br>S<br>S-W<br>W<br>N-W	Slope<br>_________ ° or<br>Level 0-20 °<br>Undulating 20-40 °<br>Rolling 40-70°<br>Moderate 70-90°<br>Steep >90°	Soil texture<br>____________<br>Gravel<br>Sand<br>Sandy loam<br>Loam<br>Clay loam<br>Clay<br>Peat<br>No soil	Soil pH<br>Acidic<br>Alkaline<br>Neutral<br>Saline<br>Yes<br>Not

Land use	Threats observed
Habitat and Site Notes *(microhabitat details, distance to agricultural crops)*	Use *(medicinal, firewood, food etc.)*
Collecting Notes *(e.g. problems encountered)*	Associated species
Plant Form	Plant Height (m)

Plant Description *(e.g. scents, flower colour, regeneration)*
